# Does the association between short-chain fatty acids and depressive symptoms vary with age? A large population-based study

**DOI:** 10.1192/j.eurpsy.2024.163

**Published:** 2024-08-27

**Authors:** R. Okubo, R. Yamamura, S. Ishikawa, T. Kimura, S. Ukawa, K. Nakamura, A. Tamakoshi

**Affiliations:** ^1^Department of Psychiatry; ^2^Division of Biomedical Oncology; ^3^Department of Public Health, Hokkaido University, Sapporo; ^4^Research Unit of Advanced Interdisciplinary Care Science, Osaka City University, Osaka; ^5^Department of Public Health and Hygiene, University of the Ryukyus, Nishihara, Japan

## Abstract

**Introduction:**

Fat plays an important role in brain function; 60% of the brain’s dry weight is fat. Among fats, omega-3 fatty acids, which are long-chain fatty acids, have been reported to reduce depressive symptoms. On the other hand, there are few studies on short-chain fatty acids (SCFAs), and those that do exist are mostly animal studies, with only a few human studies (about 100 cases). This is the first study to examine the association between fecal short-chain fatty acids and depressive symptoms on a large scale in the general population.

**Objectives:**

We examined the association of fecal SCFAs with depressive symptoms. In addition, we analyzed the associations stratified by age and examined differences in the associations.

**Methods:**

This study was conducted using data from the Dynamics of Lifestyle and Neighborhood Community on Health Study (DOSANCO Health Study). The target population was all residents of the city of Suttu, Hokkaido, Japan, excluding residents of special nursing homes (n=2638). 579 individuals (22% of the target population) aged 18 years and older who were able to measure fecal SCFA participated in this study with written informed consent. Approval was obtained from the Ethics Committee of Hokkaido University School of Medicine (15-002 and 15-045). Fecal SCFA was measured by high-performance liquid chromatography. We examined the association of fecal concentrations of SCFA subtypes (i.e., acetate, butyrate, and propionate) and total SCFA concentrations (mg/g wet weight as a continuous variable) with total Patient Health Questionnaire-9 (PHQ-9) scores using multiple regression analysis. We adjusted for age, sex, habitual exercise, total energy intake, and total dietary fiber intake. We performed additional multiple regression analyses with stratification by age group (18-59 years and 60 years or older). Two-tailed tests were used for all analyses with a significance level of P < 0.05.

**Results:**

The mean age (standard deviation) of the study participants (n=534) was 58.3 (16.0) years. Among them, 48% were 18-59 years old and 54% were female. Fecal propionate concentration was significantly associated with total PHQ-9 score (beta=0.62, p<0.01). Other SCFAs and total SCFA were not significantly associated with total PHQ-9 score. In addition, using stratification analyses by age group, the associations between fecal propionate concentration and total PHQ-9 score showed a different trend by age group (beta=0.18, p=0.62 for 18-59 years; beta=0.80, p<0.01 for 60 years or older).

**Conclusions:**

The study showed an association between higher concentrations of fecal propionic acid and higher levels of depressive symptoms. The association was particularly pronounced in older people, those aged 60 years and older. The results suggest that improving dietary habits to reduce fecal propionic acid may be effective in preventing depression in the elderly.

**Disclosure of Interest:**

R. Okubo Shareolder of: None, Grant / Research support from: A Grant-in-Aid for Scientific Research from Japan Society for the Promotion of Science (No. 22K17844), Consultant of: None, Employee of: None, Paid Instructor of: None, Speakers bureau of: Speakers bureau from Takeda Pharmaceutical Company Limited, R. Yamamura: None Declared, S. Ishikawa: None Declared, T. Kimura: None Declared, S. Ukawa: None Declared, K. Nakamura: None Declared, A. Tamakoshi: None Declared

